# Supplementary material for: Increasing adverse drug reaction reporting—How can we do better?
Source: PLoS One. 2020 Aug 13;15(8):e0235591. doi: 10.1371/journal.pone.0235591 (PMC7425892; doi:10.1371/journal.pone.0235591)
Supplement: S1 File — (DOC) [file pone.0235591.s002.doc]

**Dear colleagues,**

As part of my PhD studies in the Department of Epidemiology at Ben-Gurion University of the Negev, I am conducting a research study entitled: **Intervention Program for Changing Patterns of Reporting Adverse Drug Reactions - The Missing Link in Maintaining Medication Safety**.

Participants in the study shall be physicians and nurses working in the internal medicine wards in three hospitals in Israel ("A", "B" and "C").

The goal of this study is to examine the extent to which an intervention program may improve the level of reporting of adverse drug reactions.

There are no rights or wrong answers, rather answers that reflect your attitudes on this issue.

Analysis of the questionnaire is anonymous, and the recorded details are for the purposes of this study only. However, since we would like to ask you to fill in the questionnaire in the future, it is important that you fill in the identifying information, which will be removed from the questionnaire before it is inputted into a computer file.

I request your approval for participating in the study, and I guarantee to keep all information provided confidential and not to disclose any personal identifying information. You are participating in the study of your own free will.

Please answer the questions with the utmost candor, as the value of the study depends on candid answers.

The study questions refer to all genders.

If you encounter any problem or question, please contact me at 0577-346422, or Prof. Amalia Levy at 08-6477455 or Prof. Mati Berkowitz at 0577-345152

Thank you for your cooperation,

Miri Shchory Potlog

**Please circle the answer that best reflects your agreement with each one of the following statements.**

|  | | **Strongly disagree** |  |  |  |  |  |  |  | |  | **Strongly agree** |
| --- | --- | --- | --- | --- | --- | --- | --- | --- | --- | --- | --- | --- |
| 1 | You may notice an irregular adverse reaction from drug treatment and not report it since:  Circle an answer for each question from 1a to 1e | | | | | | | | | | | |
| 1a | I know the adverse drug reaction has already been documented by the pharmaceutical company | 1 | 2 | 3 | 4 | 5 | 6 | 7 | 8 | 9 | | 10 |
| 1b | I do not know that there is a center for reporting adverse drug reactions | 1 | 2 | 3 | 4 | 5 | 6 | 7 | 8 | 9 | | 10 |
| 1c | I am not aware of the need for reporting adverse drug reactions | 1 | 2 | 3 | 4 | 5 | 6 | 7 | 8 | 9 | | 10 |
| 1d | I don't know how to report adverse drug reactions | 1 | 2 | 3 | 4 | 5 | 6 | 7 | 8 | 9 | | 10 |
| 1e | Reporting one adverse drug reaction does not significantly contribute to the reporting mechanism | 1 | 2 | 3 | 4 | 5 | 6 | 7 | 8 | 9 | | 10 |
| 2 | I spoke with pharmaceutical companies about the possibility of adverse drug reactions with their drugs | 1 | 2 | 3 | 4 | 5 | 6 | 7 | 8 | 9 | | 10 |

**Please mark, next to the sentence, Yes, No, Don't**

|  | **Yes** | **No** | **Don't know** |
| --- | --- | --- | --- |
| 3. Have you ever reported adverse drug reactions to a national reporting center? |  |  |  |

**Background questions for statistical analysis use**

**This data will not be entered into computer files and will be kept separately from the questionnaire to maintain study anonymity.**

First name:_____________ Last name:__________________

Hospital: _______________Ward:______________________

.................................................................. ...................................................................

| 🞎 Nursing student | 🞎 Nursing Intern | 🞎 Practical Nurse | 🞎 Registered Nurse |
| --- | --- | --- | --- |
| 🞎 Medical student | 🞎 Physician Intern | 🞎 Resident physician | 🞎 Expert physician |

**Profession:**

**Year of birth:** ___________ **Gender**: Male / Female

**Country of origin**: Israel / Other, specify: ____________ Immigration year: ______

**Country of professional studies**: Israel / abroad, specify: ____________

**No.** **of Years of seniority as nurse/physician**: ___________________

**Education**: Academic degree 🞎 BA 🞎 MA 🞎 PhD **Other**: _____________

**Expertise/specialization**: 🞎 Internal medicine 🞎 Geriatrics 🞎 Cardiology

🞎 Intensive Care 🞎 Gastroenterology 🞎 Endocrinology (Diabetes)

🞎 Rheumatology 🞎 Infectious (infection prevention) 🞎 Nephrology 🞎 Oncology 🞎 Other: __________

**Additional roles you fill**: 🞎 I do not fill any additional roles

🞎 Management roles, specify: ___________ 🞎 Academic, specify: ____________

🞎 Other, specify: _____________

**Period you've worked in an internal medicine division: months ___ years___**

**Indicate your workplaces:**  🞎 Hospital 🞎 Private clinic 🞎 Community

**The number of patients you treat per day ____________**

**The Number of drugs you prescribe/ examine treatment/ dispense per day __**

**Percentage of patients under your responsibility who take more than one drug per day ________ %**

**What is the most convenient reporting channel for you?** 🞎 Phone 🞎 Fax 🞎 Email 🞎 Website

Some suggestions you would like to make regarding a program for adverse drug reactions reporting: __________________________________________________

__________________________________________________________________

__________________________________________________________________

__________________________________________________________________
