# Supplementary material for: Increasing adverse drug reaction reporting—How can we do better?
Source: PLoS One. 2020 Aug 13;15(8):e0235591. doi: 10.1371/journal.pone.0235591 (PMC7425892; doi:10.1371/journal.pone.0235591)
Supplement: S2 File — (DOC) [file pone.0235591.s003.doc]

**עמית/עמיתה יקרים**

במסגרת לימודי לתואר שלישי במחלקה לאפידמיולוגיה באוניברסיטת בן-גוריון בנגב, אני עורכת מחקר שנושאו: **תוכנית התערבות לשינוי דפוסי הדיווח על תופעות לוואי מתרופות – החוליה החסרה בשמירה על בטיחות הטיפול התרופתי.**

#### במחקר ישתתפו רופאים ואחיות העובדים בחטיבת מחלקות פנימיות, בשלושה בתי חולים בארץ ("א", "ב" ו"ג").

# מטרת המחקר לבדוק באיזו מידה תוכנית התערבות בנושא, עשויה לשפר את רמת הדיווח על תופעות לוואי מתרופות.

# אין תשובות נכונות או בלתי נכונות, התשובות ישקפו את עמדותיך בנושא.

# ניתוח השאלון אנונימי והפרטים שירשמו בו הם לצורכי מחקר זה בלבד. יחד עם זאת, מאחר שברצוננו להעביר לך את השאלון גם בעתיד, יש חשיבות במילוי הפרטים המזהים, אשר יוסרו מהשאלון לפני הקלדתו לקובץ מחשב.

# אני מבקשת את הסכמתך להשתתף במחקר ומתחייבת לשמור כל מידע שימסר בסודיות מירבית ולא לפרסם פרטים אישיים מזהים כלשהם. השתתפותך במחקר היא מרצונך החופשי.

# אנא, עני/ענה על השאלות במלואן בכנות מרבית, כי בכך תלוי ערכו של המחקר.

# בעמודים הבאים, מטעמי קיצור הפנייה היא בגוף זכר והכוונה לשני המינים.

# בכל בעיה או שאלה ניתן לפנות אלי בטלפון 0577-346422 או לפרופ' עמליה לוי בטל' 08-6477455

# או לפרופ' מתי ברקוביץ בטל' 0577-345152

# תודה על שיתוף הפעולה

# שחורי-פוטלוג מירי

**הקף בעיגול את התשובה המתאימה ביותר למידת ההסכמה שלך עם כל אחד מההיגדים הבאים.**

|  | | **מסכים בהחלט** |  |  |  |  |  |  |  |  | **כלל לא מסכים** |
| --- | --- | --- | --- | --- | --- | --- | --- | --- | --- | --- | --- |
| 1 | ייתכן שתזהה תופעת לוואי חריגה מטיפול תרופתי ולא תדווח עליה, כי:  סמן תשובה לכל אחת מ-1א-1ה | | | | | | | | | | |
| 1א | ידוע לי שתופעת הלוואי תועדה כבר על ידי חברת התרופות | 10 | 9 | 8 | 7 | 6 | 5 | 4 | 3 | 2 | 1 |
| 1ב | אני לא יודע שקיים מרכז לדיווח על תופעות לוואי מתרופות | 10 | 9 | 8 | 7 | 6 | 5 | 4 | 3 | 2 | 1 |
| 1ג | אינני מודע לצורך בדיווח על תופעות לוואי מתרופות | 10 | 9 | 8 | 7 | 6 | 5 | 4 | 3 | 2 | 1 |
| 1ד | אינני יודע כיצד לדווח על תופעות לוואי מתרופות | 10 | 9 | 8 | 7 | 6 | 5 | 4 | 3 | 2 | 1 |
| 1ה | דיווח על תופעת לוואי אחת אינה תורמת באופן משמעותי למנגנון הדיווח | 10 | 9 | 8 | 7 | 6 | 5 | 4 | 3 | 2 | 1 |
| 2 | דיברתי עם חברות תרופות על אפשרות של תופעות לוואי מתרופותיהן | 10 | 9 | 8 | 7 | 6 | 5 | 4 | 3 | 2 | 1 |

**בשאלה שלפניך אתה מתבקש לסמן**

**"כן", "לא" או "לא יודע"**

|  | **כן** | **לא** | **לא יודע** |
| --- | --- | --- | --- |
| 3. האם אי פעם דיווחת למרכז ארצי לדיווח על תופעות לוואי מתרופות? |  |  |  |

**שאלות רקע שישמשו לניתוח הסטטיסטי**

**נתונים אלה לא יוקלדו לקובצי המחשב ויופרדו מהשאלון בכדי לשמור על אנונימיות במחקר.**

שם פרטי:_____________ שם משפחה:__________________

בית חולים:_____________ מחלקה:___________________

.................................................................. ..................................................................................

| 🞎 סטודנט/ית לסיעוד | 🞎 סטאג'ר/ית לסיעוד | 🞎אחות מעשית | 🞎 אחות מוסמכת |
| --- | --- | --- | --- |
| 🞎 סטודנט/ית לרפואה | 🞎 סטאג'ר/ית לרפואה | 🞎 רופא/ה מתמחה | 🞎 רופא/ה מומחה |

**מקצוע:**

**שנת הלידה שלך**_______  **מגדר**: זכר / נקבה

**ארץ לידה:** ישראל / אחר, פרט:_______________ שנת עליה: ____________

**המדינה בה למדת את המקצוע** ישראל/חו"ל פרט :_________________

**מספר שנות ותק בסיעוד/רפואה:** _________________________________

**השכלה:** תואר אקדמי 🞎 BA 🞎 MA 🞎 MD 🞎 PhD  **אחר:_________**

**מומחיות/התמחות ספציפית:**  🞎פנימית 🞎 גריאטריה 🞎 קרדיולוגיה 🞎 טיפול נמרץ

🞎 גסטרואנטרולוגיה 🞎 אנדוקרינולוגיה (סוכרת) 🞎 ריאומטולוגיה

🞎 זיהומיות (מניעת זיהומים) 🞎 נפרולוגיה 🞎 אונקולוגיה 🞎 אחר:__________

**התפקידים הנוספים אותם אתה ממלא:** 🞎 לא ממלא תפקידים נוספים

🞎תפקידים ניהוליים, פרט:______________ 🞎 אקדמיים, פרט:______________

אחר, פרט**:______________**

**פרק הזמן שבו אתה עובד בחטיבה הפנימית: ________ חודשים ________ שנים**

**סמן את מקומות העבודה** **שלך**: 🞎 בית חולים 🞎 רפואה פרטית 🞎 קהילה

**מספר החולים שבהם אתה מטפל ביום ____________**

**מספר התרופות שאתה רושם/בוחן את הטיפול התרופתי/מחלק ביום** **__________**

**אחוז החולים שתחת אחריותך המשתמשים ביותר מתרופה אחת ביום** %________

**מהו אמצעי הדיווח הנוח ביותר עבורך?** 🞎 טלפון 🞎 פקס 🞎 דוא"ל 🞎 אתר אינטרנט

מספר הצעות שהיית רוצה להעלות לגבי תוכנית לדיווח על תופעות לוואי מתרופות:

_______________________________________________________________________________________________________________________________________________________________________________________________________________
