# Supplementary material for: Increasing adverse drug reaction reporting—How can we do better?
Source: PLoS One. 2020 Aug 13;15(8):e0235591. doi: 10.1371/journal.pone.0235591 (PMC7425892; doi:10.1371/journal.pone.0235591)
Supplement: Supplementary file 3 [file pone.0235591.s004.doc]

elbairaV		R.O	59 IC	eulav-p
ıI		1.47	1.12	100.0
F	(o	61.3	-54.1	400.0
S		23.2	-41.1	20.0
P		07.2	-13.1	700.0
