# Supplementary material for: Increasing adverse drug reaction reporting—How can we do better?
Source: PLoS One. 2020 Aug 13;15(8):e0235591. doi: 10.1371/journal.pone.0235591 (PMC7425892; doi:10.1371/journal.pone.0235591)
Supplement: Attachment — Submitted filename: Answeres plos one 26 10 19 .docx [file pone.0235591.s005.docx]

To: Karen Cohen
Academic Editor

**PLOS ONE**

**Re**: PONE-D-19-24600 “Increasing Adverse Drug Reaction Reporting – How can we do better?”

Dear Editor,

Thank you for your letter of October 5, 2019, and for the attached reviews. We have found your comments and the reviewers' comments most helpful and have addressed them in our revised version.

As for the comments:

1. When submitting your revision, we need you to address these additional requirements. Please ensure that your manuscript meets PLOS ONE's style requirements, including those for file naming.

**Answer:** The corrected manuscript meets PLOSE ONE's style requirements, including those for file naming, as requested.

2. Thank you for including your ethics statement: The study was approved by the institutional review board of each medical center respectively.

Please amend your current ethics statement to include the full name of the ethics committee/institutional review board(s) that approved your specific study.

Once you have amended this/these statement(s) in the Methods section of the manuscript, please add the same text to the “Ethics Statement” field of the submission form (via “Edit Submission”).

**Answer:** The full names of ethics statements were corrected in the "Methods" and in the "Ethics Statement" in the revised version, as suggested:

(A - Shamir (Asaf Harofe) Ethics ("Helsinki") Committee; (protocol Number 180/10) B - Haemek Medical Center - Committee Helsinki; (protocol Number EMC-0107-10) C - Kaplan Ethics ("Helsinki") Committee (protocol Number ver:1 KAP 1).

This paragraph was added to the "Methods" (page 7, line 133), and to the "Ethics Statement" field of the submission.

3. Please include additional information regarding the survey or questionnaire used in the study and ensure that you have provided sufficient details that others could replicate the analyses. For instance, if you developed a questionnaire as part of this study and it is not under a copyright more restrictive than CC-BY, please include a copy, in both the original language and English, as Supporting Information.

**Answer:** The questionnaire used in this study, both in Hebrew and translated to English are provided as "Supporting Information" in the revised manuscript, as suggested.

**להוסיף באנגלית: מצורפים רק החלקים בשאלון שבהם השתמשתי למאמר זה ??**

4. Please provide additional details regarding participant consent. In the ethics statement in the Methods and online submission information, please ensure that you have specified (1) whether consent was informed and (2) what type you obtained (for instance, written or verbal, and if verbal, how it was documented and witnessed). If consent was implied due to the completion and returning of the questionnaire, please state this in the Methods section

**Answer**: In the introduction of the questionnaire, a detailed explanation regarding the research and its rational was provided (See Supplement 1 in the Supporting Information). In this section, the participants' consent to be included in the research, was asked. A verbal consent was received during a meeting in which the research was presented to each of the participants. This information was added to the "Methods" section (page 6, line 101) of the revised manuscript, as recommended:

In the introduction of the questionnaire, a detailed explanation regarding the research and its rational was provided (See Supplement 1 in the Supporting Information). In this section the participant consent to be included in the research was asked. A verbal consent was received during a meeting in which the research was presented to each of the participant.

4. Please include your tables as part of your main manuscript and remove the individual files. Please note that supplementary tables (should remain/ be uploaded) as separate "supporting information" files**להוציא בהטענה את הטבלאות**

**Answer**: All the Figures and the Tables were implemented as part of the main manuscript, as suggested.

Figure 1 – Page 10, line 211

Table 1 – Page 11, line 232

Table 2 – Page 12, line 244

Table 3 – Page 13, line 257

Table 4 – Page 15, line 273

Table 5 – Page 16, line 290

Table 6 – Page 17, line 303

5. Please amend the manuscript submission data (via Edit Submission) to include authors Lee H. Goldstein, Lidia Arcavi, Renata Shihmanter, Matitiahu Berkovitch and Amalia Levy.

**Answer:** Thank you for the remark. This was done in the revised version, as recommended.

6. Please amend either the abstract on the online submission form (via Edit Submission) or the abstract in the manuscript so that they are identical.

**אותו אבסטרקט במאמר ובהטענה אחרי העורכת לשונית**

**Answer:** Thank you for the remark. This was performed in the revised manuscript, as suggested.

7. We note that you have indicated that data from this study are available upon request. PLOS only allows data to be available upon request if there are legal or ethical restrictions on sharing data publicly.

**Answer:** The current study is not a clinical one. As such, the data is consisted of demographic details and opinions regarding drug adverse reaction reporting. There are no legal or ethical restrictions on sharing data publicly.

a) If there are ethical or legal restrictions on sharing a de-identified data set, please explain them in detail (e.g., data contain potentially identifying or sensitive patient information) and who has imposed them (e.g., an ethics committee). Please also provide contact information for a data access committee, ethics committee, or other institutional body to which data requests may be sent.

**Answer:** There are no ethical or legal restrictions on sharing a de-identified data set.

As for the reviewers' comments:

**Reviewer #1**

- "Thank you for the opportunity to review and comment on your research. I am aware of the amount of work done and my comments aim at helping you improve your manuscript. The first thing that surprises me is that your research dates from 2013 and 6 years have elapsed since then, some statements you make (see below) might be out of date.

The references used in the introduction are old/very old: 81-84: "...new legislation was recently launched...2014" or "the reporting rate in Israel...is still very low", this with a reference from 1999. While underreporting might still be a problem, I would be very surprised that nothing has changed in the past 20 yrs. Please update with actual recent figures**."האם צריך להתייחס שא מצאנו תמונות ?**

**Answer:** Newer references were used in the revised manuscript, as recommended. This information was added to the "Introduction" (Page 5, Line 93) of the revised version and it now appears as follow:

The Israeli ministry of health (MOH) published at August 2012 guidelines for reporting adverse events and new safety information. This document specifies the types of information requiring reporting by the Marketing Authorization Holder (MAH) and enables the MAH to appoint a pharmacist, who should serve as a qualified Person responsible for in matters related to the reports included in this SOP, according to the standard worldwide practice. Those regulations were update at February 2013 in order to clarify the work processes related to reporting ADRs and new safety information, to update the definitions of the SOP and to update the types of information requiring reporting by the MAH. The aim of additional update from May, 2013 was to adjust the SOP to the Pharmacists Regulations (16). A new regulation was launched at October, 2014, in which a reporting system in medical institutions for both common and severe ADRs was established. On July 2019, a new portal for reporting adverse events to the Risk Management Department has emerged.

Data regarding the low reporting rate in Israel was updated - Page 5, line 109, ref.18

Schwartzberg el al., identified 16,409 of Individual Case Safety Reports (ICSRs) submitted to the MOH’s ADRs central database between September 2014 and August 2016. However, of these reports, only 5.5% were submitted by health care professionals from medical institutions, while 94.3% were submitted by pharmaceutical companies (MAH and importers) and only 0.2% of the reports have been submitted by patients and the general public [18].

- "Methods:

133-142: the answers to the question with "because" do not make sense in the context of the questions, while valid as stand alone. Please clarify."

**Answer:** This question refers to the reasons for not reporting after identifying adverse reaction effects of drug therapy. It contains 5 possible causes for not reporting. The word “because” has been replaced by the word “since”- Page 9, line 175.

- "To the reader it is not clear why the focus was on Hebrew (obvious) and Russian speakers (much less obvious). Please explain."

**Answer:** In Israel, a high percentage of physicians and nurses are from the Commonwealth States and Russian is their first language. In order to make it easier for them to complete the questionnaire and to increase the response rate it was translated to Russian. This explanation was added in the revised version, as suggested (Page 7, line 125).

- "143-148: you mention two statements analyzed, but B is actually a question. Please clarify."

**Answer:** The word “statements” has been changed to “items” – Page 10, line 190.

- "Also, behavior change cannot be measured by subjective statements. In this case behavior change can be measured by the number of reports submitted before and after the intervention."

**Answer:** Indeed, as stated by the reviewer, behavior change cannot be measured by subjective statements. However, it could be measured by the number of reports submitted before and after the intervention. This is presented in the data of Figure 1, where a rapid and substantial increase in the number of ADR reports was noted in the intervention group during the intervention period while almost no change in the number of ADRs reports was observed in the control group. This information is presented in the "Discussion" of the revised manuscript, as recommended, and it now appears: (Page 19, line 344)

The trend presented in the data that the rate of reports decreases over time after the intervention program is discontinued implies that continued intervention may be able to maintain the high rate of reports. Interestingly, the reporting rate in the control group was higher than in the intervention group at base line, though it was not statically significant. The intervention plan increased the reporting rate and the differences between the control and the intervention went up and were statically significant all along the intervention period. However, as you move away from the intervention period, the amount of reports decreases till it reached to the trend that was observed at the baseline.

- "The statistical analysis of the results of the questionnaires is very detailed but unfortunately does not lead to conclusions on how to improve future interventions."

**Answer:** According to the statistical analyzed data, the increase in the rate of ADRs in the intervention group was noted during the 5-month intervention period. Nevertheless, it was decreased toward the end of the study. Therefore, it was suggested that the intervention program, as well as training and educating the medical practitioners should be maintained. This was addressed to the conclusion (page 23, line 422) and Abstract (page 3, line 52).

Maintaining the intervention program could be carried out by nomination of a pharmacovigilance specialist trustee that will run an intervention program routinely. This expert could be a physician, a nurse or a pharmacist. Visits, personal discussions, posters, lectures about the importance of ADRs reporting and how to perform it and sending text messages to the medical staff on their mobile phones with reminders and relevant information should be used in order to continuously raise their awareness reporting about ADRs. In addition, the personal contact of the medical staff with the trustee will encourage their commitment to report about ADRs.

Thus, implementation and maintenance of an intervention program continuously, by a pharmacovigilance specialist personal staff member, will improve their ADRs reporting rates.

- "Results: You write that the reporting in the intervention group was higher than in the control and that the effect diminished over time. What you do not mention is that according to Fig 1, the reporting in the intervention group went nearly back to baseline and was even lower than in the control group."

**Answer:** Indeed as the reviewer commented, although the reporting in the intervention group was lower than that of the control group, it was not statically significant. The following sentence was added to the revised version, as suggested and it appears: duration . After the intervention period the reporting rate in the intervention group went nearly back to baseline and was lower than the control group at the same time as it was at base line. (Page 12, line 243)

- "While the single values might not reach statistical significance, the trend is still impressive and must be mentioned and discussed - not doing so misleads the reader. Please rectify (179-183)."

**Answer:** This information was added to the "Discussion" of the revised manuscript page 20, line 344.

The trend presented in the data that the rate of reports decreases over time after the intervention program is discontinued implies that continued intervention may be able to maintain the high rate of reports. Interestingly, the reporting rate in the control group was higher than in the intervention group at base line and at the end of the study, though it was not statically significant. The intervention plan increased the reporting rate and the differences between the control and the intervention went up and were statically significant all along the intervention period. However, as you move away from the intervention period, the amount of reports decreases till it reached to the trend that was observed at the baseline.

- "Discussion:
  see comment above"

**Answer:** The discussion section was amended according to the comment – page 20, line 344

- "Conclusion:
  see above and please explain more in detail how interventions can be improved based on your data and their analysis."

**Answer:** The increase in the rate of ADRs in the intervention group was noted during the 5-month intervention period. Nevertheless, it was decreased toward the end of the study. Therefore, it was suggested that the intervention program, as well as training and educating the medical practitioners should be maintained.

**ולתקן את התשובה ואז הציטוט נכון	לשפץ**

page 23, line 422

Maintaining the intervention program could be carried out by nomination of a pharmacovigilance specialist trustee that will run an intervention program routinely. This expert could be a physician, a nurse or a pharmacist. Visits, personal discussions, posters, lectures about the importance of ADRs reporting and how to perform it and sending text messages to the medical staff on their mobile phones with reminders and relevant information should be used in order to continuously raise their awareness reporting about ADRs. In addition, the personal contact of the medical staff with the trustee will encourage their commitment to report about ADRs.

- "Language: the manuscript would benefit from a revision by a native English speaker."

**Answer:** The manuscript was reviewed by a professional native English speaker.

**Reviewer #2:**

- "Methods section: description (grade) is not very clear. Scoring and description seem to contradict. Should be rephrased."

**Answer:** Thank you for this very correct note. The word “grade” has been replaced by the word “score” all over the text and in the tables.

- "Inconsistent/non standardized/interchangeable use of the terms side effects, adverse effects, adverse reactions."

**Answer:** The terms side effects and adverse effects were changes to "adverse reactions" all over the text.

- "Pharmacovigilance is described as a pharmacy practice. I am not aware of any such categorization."

**Answer:** Thank you for the remark. The definition of Pharmacovigilance by the WHO was introduced into the text of the revised version, as suggested– Page 4, line 76.

Pharmacovigilance is defined by the WHO as the science and activities relating to the detection, assessment, understanding and prevention of adverse reactions or any other drug-related problem

- "How were duplicate reports identified/handled (since for Group A, post intervention reports came both from the facility and the MOH website."

**Answer:** The reports were checked and there were no duplicate reports by staff members on both reporting channels in the intervention group. This information was added to the revised manuscript, as recommended (Page 11 Line 229)

The reports were checked and there were no duplicate reports by the staff members on both reporting channels in the intervention group.

- "Interventions such as these are known to have a temporary positive impact on reporting. What is more important is to discuss how to sustain this. Also, will such an intervention lead to quality improvements?"

**Answer:** This issue was already discussed –page 23, line 422

- "Authors have explained that there is no data repository. And that primary data could be provided as email attachments."

**Answer:** All needed data is presented.

Sincerely,
