# Supplementary material for: Increasing adverse drug reaction reporting—How can we do better?
Source: PLoS One. 2020 Aug 13;15(8):e0235591. doi: 10.1371/journal.pone.0235591 (PMC7425892; doi:10.1371/journal.pone.0235591)
Supplement: Attachment — Submitted filename: Response to Reviewers.docx [file pone.0235591.s006.docx]

To: Karen Cohen
Academic Editor

PLOS ONE

Re: PONE-D-19-24600R1 “Increasing Adverse Drug Reaction Reporting – How can we do better?”

Dear Editor,

Thank you for your letter of March 2, 2020, and for the attached reviews. We have found your comments and the reviewers' comments most helpful and have addressed them in our revised version.

As for the comments:

**Comments to the Author**

1. If the authors have adequately addressed your comments raised in a previous round of review and you feel that this manuscript is now acceptable for publication, you may indicate that here to bypass the “Comments to the Author” section, enter your conflict of interest statement in the “Confidential to Editor” section, and submit your "Accept" recommendation.

Reviewer #1: All comments have been addressed

Reviewer #3: (No Response)

Reviewer #4: (No Response)

2. Is the manuscript technically sound, and do the data support the conclusions?

The manuscript must describe a technically sound piece of scientific research with data that supports the conclusions. Experiments must have been conducted rigorously, with appropriate controls, replication, and sample sizes. The conclusions must be drawn appropriately based on the data presented.

Reviewer #1: (No Response)

Reviewer #3: Partly

Reviewer #4: Yes

3. Has the statistical analysis been performed appropriately and rigorously?

Reviewer #1: (No Response)

Reviewer #3: No

Reviewer #4: Yes

4. Have the authors made all data underlying the findings in their manuscript fully available?

The [PLOS Data policy](https://smex12-5-en-ctp.trendmicro.com:443/wis/clicktime/v1/query?url=http%3a%2f%2fwww.plosone.org%2fstatic%2fpolicies.action%23sharing&umid=0f089f87-89e2-40a8-8bb1-51f8567e8813&auth=33b0ce5d398fbfcf42d664f7d8e15051d5ed97f8-ba7627337e0f2dcb88c69367213711db610827d6) requires authors to make all data underlying the findings described in their manuscript fully available without restriction, with rare exception (please refer to the Data Availability Statement in the manuscript PDF file). The data should be provided as part of the manuscript or its supporting information, or deposited to a public repository. For example, in addition to summary statistics, the data points behind means, medians and variance measures should be available. If there are restrictions on publicly sharing data—e.g. participant privacy or use of data from a third party—those must be specified.

Reviewer #1: (No Response)

Reviewer #3: Yes

Reviewer #4: Yes

5. Is the manuscript presented in an intelligible fashion and written in standard English?

PLOS ONE does not copyedit accepted manuscripts, so the language in submitted articles must be clear, correct, and unambiguous. Any typographical or grammatical errors should be corrected at revision, so please note any specific errors here.

Reviewer #1: (No Response)

Reviewer #3: Yes

Reviewer #4: Yes

6. Review Comments to the Author

Please use the space provided to explain your answers to the questions above. You may also include additional comments for the author, including concerns about dual publication, research ethics, or publication ethics. (Please upload your review as an attachment if it exceeds 20,000 characters)

Reviewer #1: (No Response)

Reviewer #3: From the data presented and the statistical input that was implemented the investigators have concluded that. an increase in the number of ADR reports was noted in the intervention group during the intervention period, the changes in the "knowledge related to behavior” and in the "behavior related to reporting" score was significantly higher in the intervention group. Also specialist physicians and nurses, fulfilling additional positions and those working in other places demonstrated a high rate of report.

The data analysis approach is fairly routine and appears to be sufficient. Also the results are striking. However, the study lacks a rigorous study design justification. Three institutions were randomized, but the unit of analysis is the individual. There is no mention or investigation of the intraclass correlation in this type of setting and how that could impact on the varied analyses and the impact on the type I error. There is no central hypothesis.

**Answer:**

The research groups were compared in terms of background characteristics and were found to be similar in most variables, so there is no reason to assume there is an Intraclass correlation. The main hypothesis: after the intervention program there would be more ADRs reporting among medical professionals (physicians and nurses) in the intervention group compared to control group and to ADRs reporting base line. This was added to the methods section (page 10, line 204)

Other statistical issues include:

1. The multiple comparison testing issue is totally ignored, especially in Tables 3 and 4.

**Answer:**

The variables compared in Tables 3 and 4 include two groups, thus there is no need to correct for multiple comparisons. The p value of all the variables with 3 groups were found to be not significant so there is no need to correct for multiple comparison.

2. Tables 5 and 6 would suffice for a descriptive analysis. However, where is the institution effect in these tables?

**Answer:**

Indeed, beyond the intervention there could be also the influence of the medical institution itself. However, there were no significant differences between the participants from the various medical centers with regard to the basic demographic characteristics.

This information was added to the results section page 11 line 236: "433 (81.5%) medical staff members, physicians and nurses, completed the questionnaire twice, before and after the intervention. 47.8% of the participants were from the "A" medical center, 28.4% from "B" and 23.8% from "C". Distribution by gender was 69.1% females and 30.9% males. 73% were nurses and 27% physicians. No selection bias was found between the staff members completed the questionnaire the first time and those who completed it twice. No differences in personal or professional variables were found between the intervention group ("A" medical center) and the control group ("B" and "C" medical centers), except for the ratio between physicians and nurses and the subjects country of origin and average age."

3. There is insufficient tabular description of the number of cases, specialties, etc. within each of the institutions.

**Answer:**

This data was added to the results section of the revised manuscript, page 11 line 236.

4. One of the key points mentioned by the investigators is “Education and training of medical staff in good pharmacovigilance practice is necessary for adequate ADR reporting and for the reduction of ADR rates and lower the costs in the healthcare system”. There is no analysis or meaningful discussion of costs considerations.

**Answer:**

Thanks for the comment. Since reduction of costs due to ADRs reporting was not the scope of this study, we removed this section from the revised manuscript.

5. Reporting tools were conveniently telephone or online, but there is no validation of responses.

**Answer:**

This was one of the questions that was asked in the questionnaire. "ADRs were reported through the Ministry of Health computerized website (for both the intervention and the control group) or documented in binders available only in the departments of the intervention group." (Page 9, line 171). Therefore, it seems that there is no need to validate it.

The investigators would do well to have this manuscript thoroughly edited by a statistician.

**Answer:**

A statistician reviewed the revised manuscript, as suggested.

Reviewer #4: I really appreciate reading your work. I feel that the results are rather interesting. The employed methods are sound and the data are rich of info. I have just minor comments.

1. T-tests, as well as any other tests, must satisfy some assumptions. These assumptions are overlooked in the analysis. Please, provide evidence that the assumptions behind the t-test are fulfilled, to avoid misleading conclusions. Non-parametric tests could be used instead.

**Answer:**

Thank you for your remark. In the "Methods" section, there is a section of data analysis in page 10, line 209: "Means and standard deviation were used for continuous variables and examined by T or One-Way ANOVA/Mann Whitney tests based on the variables distribution." We added that: "The score of "Knowledge related to behavior" and the score of "behavior related to reporting" didn’t distribute normally, thus we used a- parametric test (Mann Whitney). Page 10 line 2011.

2. The use of regressions is sound. Nevertheless, to be confident on the obtained results, the authors must check for the Gauss-Markov assumptions. Inference on regression parameters is valid if and only if those assumptions are fulfilled. Moreover, I am wondering if multicollinearity may be an issue. Please, extend the regression model to include interactions. In general, the analysis of residuals may be included. It would be very useful to evaluate the reliability of the results.

**Answer:**

Thank you for your Suggestion. We have checked again for Gauss-Markov assumptions and the Inference on regression parameters was found valid and there was no multicollinearity and interactions between the independent variables.

7. PLOS authors have the option to publish the peer review history of their article ([what does this mean?](https://smex12-5-en-ctp.trendmicro.com:443/wis/clicktime/v1/query?url=https%3a%2f%2fjournals.plos.org%2fplosone%2fs%2feditorial%2dand%2dpeer%2dreview%2dprocess%23loc%2dpeer%2dreview%2dhistory&umid=0f089f87-89e2-40a8-8bb1-51f8567e8813&auth=33b0ce5d398fbfcf42d664f7d8e15051d5ed97f8-f10bfce45ccf0aa285f7fb62a865fd58464497ad)). If published, this will include your full peer review and any attached files.


If you choose “no”, your identity will remain anonymous but your review may still be made public.


**Do you want your identity to be public for this peer review?** For information about this choice, including consent withdrawal, please see our [Privacy Policy](https://smex12-5-en-ctp.trendmicro.com:443/wis/clicktime/v1/query?url=https%3a%2f%2fwww.plos.org%2fprivacy%2dpolicy&umid=0f089f87-89e2-40a8-8bb1-51f8567e8813&auth=33b0ce5d398fbfcf42d664f7d8e15051d5ed97f8-dd9a870a82a032732c388ee575c7c2936963ea1b).

Reviewer #1: No

Reviewer #3: No

Reviewer #4: No

Sincerely,

Miri Potlog shchory
