# Supplementary material for: Increasing adverse drug reaction reporting—How can we do better?
Source: PLoS One. 2020 Aug 13;15(8):e0235591. doi: 10.1371/journal.pone.0235591 (PMC7425892; doi:10.1371/journal.pone.0235591)
Supplement: Attachment — Submitted filename: Response to Reviewers.docx [file pone.0235591.s007.docx]

To: Karen Cohen
Academic Editor	PLOS ONE

Re: PONE-D-19-24600R2
Increasing Adverse Drug Reaction Reporting - How can we do better?
PLOS ONE

Dear Editor,

Thank you for your letter of May 12, 2020, and for the comments. We have found your comments most helpful and have addressed them in our revised version.

**As for the comments:**

"It is essential that the following points be addressed:

- Please can you provide some information as to how the 3 sites were allocated to either "intervention" or "control" The reviewer's comment re site-specific factors has not been addressed. There may be differences between the sites that contribute to the differences seen, that are not captured in the variables presented in the manuscript. Please address this comment more comprehensively, and include this in the limitations"

**Answer:**

Three medical centers have participated in the study (Center "A", "B" and "C"). "The randomization among the centers was raffled by an external person who was not related to the study." (Page 7, lines 131-132). "Medical center "A" was randomly selected to be the intervention group. Medical centers "B" and "C" were merged and served as the control group" (Page 7, lines 129-131). "The medical centers selected for the study were public hospitals serving an urban and rural population of 0.5-1 million people each." (Page 7, lines 123-124). The participants were from the: "internal medicine divisions from three public medical centers in Israel: "A", "B" and "C", while each division served as a cluster." (Page 7, lines 121-123). This information is presented in the revised manuscript, as suggested.

- "Please include description of the model building strategy for multivariable models. How were the included variables chosen for the multivariable models presented? Are there any other variables included in these models? If yes please specify."

**Answer:**

The following paragraph was added to the method section (page 11 lines 231-237): "The building strategy for multivariable models was forced all the independent variables to one block. Both statistical and clinical justifications were considered. The models included all the variables that were found to be significant (p<0.05) in the univariate data analysis, and covariates that were important to controlling for, as a baseline characteristics, according to the research questions. All the independent variables that were included in the analyses were presented in the results of the multivariable models."

_____________________________________________________________________

- "The discussion and conclusion does not adequately address the fact that the impact of the intervention waned with time. It is therefore by no means certain that this intervention would improve safety of medicine use in the long term. There is little basis for reaching the conclusion that this intervention would lead to "overall reduced health costs"- suggest remove this statement from the conclusion.
  Please add a paragraph on the limitations of this study. This should include the small number of sites included."

**Answer:**

It is mentioned in the discussion section that: "The trend presented in the data that the rate of reports decreased over time after the intervention program was discontinued implies that continued intervention may be required to maintain the high rate of reports." (Page 20 lines 377-380) and also that: "However, as distance from the intervention period increased, the amount of reports decreased eventually reverting to the trend that was observed at the baseline. (Page 21 lines 384-386)

We also added that: "The impact of the intervention waned with time. An example to this trend was shown in an educational intervention program to improve physicians' reporting of ADRs. In this study the reporting rate in the intervention group increased during the intervention, while it gradually decreased through 13 months of follow-up. [9]." (Page 21 lines 386-390)

"Due to the observation that the reporting rate decreased with time upon the finalization of the intervention period it seems that….." This was added to the conclusions section (Page 24, lines 458-460)

We agree that: "It is therefore by no means certain that this intervention would improve safety of medicine use in the long term and also reduce health costs". As suggested, this statement was removed from the conclusion.

We added a paragraph on the limitations of the study, as suggested. (Pages 24-25 lines 476-486) **Limitations**

The study was carried out in internal divisions of only 3 public medical centers, and therefore may not have an external validation in other hospitals.

The study included only physicians and nurses, therefore the results cannot be applied to additional medical professionals.

There were some differences in the basic characteristics between the clusters (hospitals) which may have affected the quality of the intervention to a certain extent. However, after adjusting for the demographic variables, we can assume that the results of the study are indeed due to the effect of the intervention.

We cannot assume from this study that the effect of the intervention would improve safety of medicine use in the long term and reduce health costs.
